# Cholera risk in cities in Uganda: understanding cases and contacts centered strategy (3CS) for rapid cholera outbreak control

**DOI:** 10.11604/pamj.2021.39.193.27794

**Published:** 2021-07-12

**Authors:** Godfrey Bwire, John Baptist Waniaye, Julius Simon Otim, David Matseketse, Atek Kagirita, Christopher Garimoi Orach

**Affiliations:** 1 Department of Integrated Epidemiology, Surveillance and Public Health Emergencies, Ministry of Health, Kampala, Uganda,; 2 Department of Emergency Medical Services, Ministry of Health, Kampala, Uganda,; 3 Directorate of Public Health, Kampala Capital City Authority, Kampala, Uganda,; 4 UNICEF, Emergencies Programme, Kampala, Uganda,; 5 Uganda National Health Laboratory Services/Central Public Health Laboratories, Ministry of Health, Kampala, Uganda,; 6 Department of Community Health and Behavioral Sciences, Makerere University College of Health Sciences, School of Public Health, Kampala, Uganda

**Keywords:** Cholera, outbreak, prevention, Africa, treatment, chemoprophylaxis, infection, Uganda, city, Kampala

## Abstract

**Introduction:**

in the recent past, cities in sub-Saharan Africa have reported serious cholera outbreaks that last for several months. Uganda is one of the African countries where cities are prone to cholera outbreaks. Studies on cholera in Bangladesh show increased risk of cholera for the immediate household members (contacts) yet the control interventions mainly target cases with little or no focus on contacts. This study aimed to describe the rapid control of cholera outbreaks in Kampala and Mbale cities, Uganda, using, “Cases and Contacts Centered Strategy (3CS)” that consisted of identification and treatment of cases, promotion of safe water, sanitation, hygiene (WaSH) and selective chemoprophylaxis for the contacts.

**Methods:**

a cross-sectional study was conducted in 2015-2016 in the Kampala and Mbale cities during cholera outbreaks. Cholera cases were treated and 816 contacts from 188 households were listed and given cholera preventive packages. Data were collected, cleaned, analysed and stored in spreadsheet. Comparison of categories was done using Chi-Square test.

**Results:**

a total of 58 and 41 confirmed cholera cases out of 318 and 153 suspected cases were recorded in Kampala and Mbale cities respectively. The outbreaks lasted for 41 days in both cities. Case fatality rates were high; 12.1% (5/41) for Mbale city and 1.7% (1/58) for Kampala city. Fifty-five percent (210/379) of stool samples were tested by culture to confirm V. choleraeO1. No contacts listed and given cholera preventive package developed cholera. Both sexes and all age groups were affected. In Kampala city, the males were more affected than the females in the age groups less than 14 years, p-value of 0.0097.

**Conclusion:**

this study showed that by implementing 3CS, it was possible to rapidly control cholera outbreaks in Kampala and Mbale cities and no cholera cases were reported amongst the listed household contacts. The findings on 3CS and specifically, selective antibiotic chemoprophylaxis for cholera prevention, could be used in similar manner to oral cholera vaccines to complement the core cholera control interventions (disease surveillance, treatment of cases and WaSH). However, studies are needed to guide such rollout and to understand the age-sex differences in Kampala city.

## Introduction

Cholera a preventable bacterial disease that is a major cause of morbidity and mortality in developing countries [1]. In the recent past, several cities in sub-Saharan Africa have experienced severe cholera outbreaks with associated socioeconomic consequences [2-4]. In addition to reporting a big number of cholera cases, some of these city outbreaks last for several months. For instance, it took almost two years (January 2017 to November 2018) to control the cholera outbreak in Kinshasa city, Democratic Republic of Congo [5] and in Lusaka city, Zambia cholera outbreak was controlled after eight months (October 2017 to May 2018) [3].

Just like the other countries in sub-Saharan Africa region, Uganda is prone to infectious diseases outbreaks especially cholera and Ebola [6,7]. The first cholera outbreak in Uganda occurred in 1971 and since then the country has continued to report cholera cases [8-11]. In the last two decades, Uganda reported cholera outbreaks almost every year [6,12]. Propagation of cholera in Uganda is driven by the favourable environmental conditions characterised by inadequate access to safe water and sanitation [13-16]. Cholera control in Uganda is guided by the national cholera prevention guidelines [17]. These guidelines are elaborate on surveillance, patient care, Water Sanitation and Hygiene (WaSH) interventions, infection control and health education. However, unlike in Ebola Virus Disease (EVD) outbreaks in which contacts are listed and followed up daily, in cholera, the focus is on treatment of the sick (those with symptoms and signs) while the contacts are only given health education for infection prevention. Cholera contacts are not listed yet studies shown high risk of infection of cholera amongst the households members of cholera cases/patients [18].

In 2015, Uganda Meteorological Authority forecasted that the *El Nino* rains would fall starting in September 2015 and end in February 2016. Previous *El Nino* rains in Uganda were associated with widespread cholera outbreaks and disruption of socioeconomic activities [8,19,20]. Therefore, the government of Uganda promoted and explored strategies to limit cholera outbreaks as part of *El Nino* preparedness. The good past lessons such as contact tracing that had ensured successful EVD outbreaks control in Uganda were embraced [7]. The Ministry of Health strengthened preparedness for cholera prevention as per the national cholera prevention and control guidelines [17] by promotion of surveillance (identification and reporting of cholera cases and suspected ones) and treatment of cases, safe water, sanitation and hygiene promotion.

**Figure 1:**
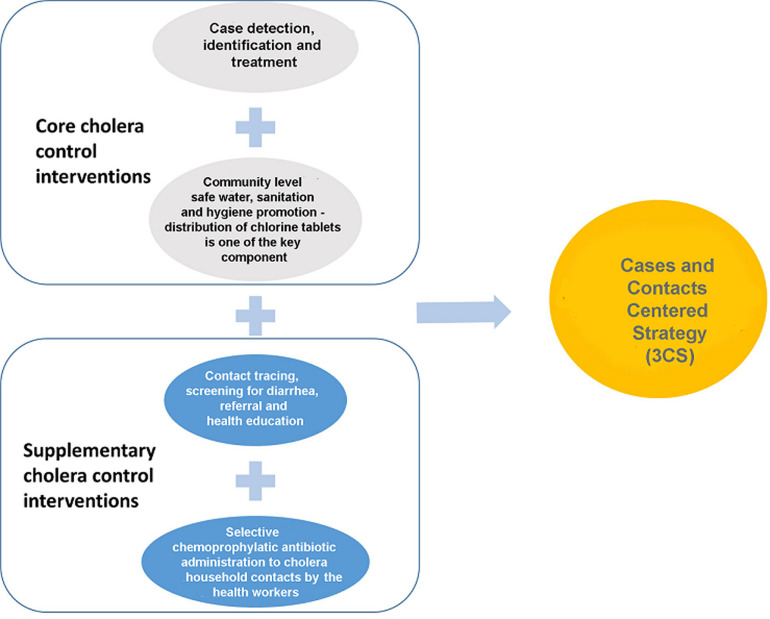
the components of cases and contacts centered strategy that were used to achieve rapid control of the cholera outbreaks in Kampala and Mbale cities in the period 2015-2016

During the last quarter of 2015, cholera outbreaks were detected in Kampala and Mbale cities. Investigations were carried out by the Ministry of Health (MOH) and the outbreaks confirmed. Following the confirmation of cholera outbreaks in the two cities of Kampala and Mbale a new strategy was adopted that incorporated the lessons learnt from previous EVD outbreak management [21,22]. The new strategy for cholera control consisted of a package of core interventions plus complementary ones namely, contact tracing and education, selective chemoprophylaxis and distribution of chlorine tables as shown in Figure 1. This paper aims to shares the lessons learnt from the cases and contacts centered strategy (3CS) for cholera control in cities in Uganda so as to recommend its use in similar settings in sub-Saharan Africa in order to accelerate the attainment of the World Health Organization cholera elimination Roadmap target by 2030 [23].

## Methods

**Study design and setting:** a cross-sectional descriptive study was conducted in Kampala and Mbale cities during the cholera outbreaks of 2015-2016. These cities were purposively selected because the bore high risk of cholera outbreak occurrence due to presence of informal settlements (slums) that were characterised by low access to WaSH.

**Study population:** the first category of the study population was the confirmed and suspected cholera cases that were located in Kampala and Mbale cities in the study period, 2015-2016. The second category of the study population was the contacts of the cholera cases (household members) that were present during the study period.

**Definitions:** the authors used the MOH national cholera guidelines to categorize cholera cases and contacts [17]. In addition, they used the Uganda Bureau of Statistics standard to define the households [24]. The definitions for study participants were as follows:

*A suspected cholera case:* in an area where cholera outbreak has not been declared, any patient age 5 years or more, presenting with dehydration or a death from acute watery diarrhoea or in an area with declared epidemic, any person age 2 years or more with acute watery diarrhoea.

*A confirmed cholera case:* a suspected case in which *Vibrio cholerae (V. cholerae)* serogroup O1 or O139 has been isolated in the stool.

*A cholera contact:*a person sharing the same household with the suspected cholera case or confirmed case. In this study, the authors did not restrict the definition of contacts to the persons sharing a household with a confirmed cholera case because not all cases had laboratory stool samples tested by culture (a confirmatory test for cholera).

*A household:* a household was defined as, “a group of persons who normally live and eat together” [24].

### Data collection

*Study variables:* the information collected on cholera suspects and cases included the: place of residence, age, sex, date of onset of symptom and number of persons living within a household, telephone numbers of the cases and contacts, type of treatment given, number of contacts in the household, number of contacts followed up within the last 24 hours, outcome of treatment (survived or died), number of persons in a household with similar symptoms, number of contacts and households given the cholera preventive package (selective chemoprophylaxis, chlorine tablets, health education materials [brochure in English/local language of the area] and inspection of latrines).

*Confirmation and declaration of the outbreaks:* the outbreaks were confirmed based on the national cholera prevention guidelines [17]. After confirmation of the outbreaks the teams (health staff in the two cities) were oriented on the new strategy, 3CS for cholera control that targeted cases and their contact at both health facility and household level to control the outbreak.

*Cholera contact tracing:*follow up of cases and suspected cholera cases to their households was done by a local team of health workers. A follow-up team consisted of 2-3 persons (officers) of which one was a clinician and the other a health educator or WaSH expert. The clinician was responsible for case identification, referral of cases and for selective (targeted) administration of antibiotic chemoprophylaxis to the cholera contacts. The WaSH expert inspected the homes and provided the messages/items for cholera prevention including home management and timely referral of the sick persons. On a daily basis, the teams were issued with updated information from the cholera treatment centres (*Mulago* hospital and *Namatala* health centre). It should be noted that selective chemoprophylaxis was given within the 24-72 hours of listing of a contact and omitted if not carried out within seven days. On arrival at the affected village the team looked for the community health workers, Village Health team (VHT) [25] who guided them to the households. After identifying the household, each contact was given a single dose of *doxycyline 300mg*tablets or *ciprofloxin 1000mg* for 3 days for persons aged 12 years and above. The children, breast feeding mothers and the pregnant women were given tablets of *erythromycin, 25 mg/Kilogram body weight* in four divided doses for three days. The first medicine dose for each contact was given in the presence of the team members. The rest of the medication doses were administered by the mothers or guardians following the instructions given by the study team. Patients with diarrhoea were assessed for dehydration and other illnesses and referred for further management to the cholera treatment centres or appropriate health facilities.

**Laboratory analysis:** fresh stool samples were collected by the health workers from suspected cholera cases before administration of the appropriate antibiotics. The rectal swabs from the cholera cases were inserted in the containers containing Cary Blair transport media and delivered to the microbiology laboratory within 24 hours of stool collection. The stool samples were tested by culture and serology to determine the microorganisms present therein.

**Statistical analysis:** data were collected, entered, cleaned and stored on the spreadsheet. Data were analyzed to get frequencies, proportions (case fatality rates, attack rates). Comparison of categories was done by Chi-Square test. Spatial-temporal distribution of cases was done by use of ArcGIS software, version 10.2, licenced (ESRI, Redlands, California, USA). The figures were created using Microsoft PowerPoints, Version 2016 (Microsoft, Redmond, Washington, USA).

**Ethical considerations:** permission to conduct this study was granted by the MOH. The study was intended to assist with the public health prevention and control of cholera by the MOH and as such is Institutional Review Board exempted. Verbal informed consents were sought from the study participants (cases and contacts). Personal identifying information were collected for the purpose of identification of the contacts and follow up only. Confidentiality and privacy were observed at all stages of the study by sharing aggregated or anonymous data.

## Results

**Description of the cholera outbreaks and laboratory test results:** a total of 473 suspected cholera cases and 11 deaths were reported in Kampala and Mbale cities. Kampala city reported more cases and deaths than Mbale city. A total of 58 cases were confirmed in Kampala city out of the 318 suspected cases and in Mbale city 41 cases were confirmed out of 153 suspected cases *Vibrio cholera* O1 were repeatedly isolated from stool samples of the cases in both cities. Overall, 379 stool samples were collected from suspected cases before administration of antibiotics and subjected to culture test. Fifty-five percent (210/379, 55.4%) of these stool samples were tested by culture to confirm *V. cholera* O1. *Vibrio cholera* O1 were isolated in 58/167, 35% in Kampala city and in 37/98, 37.8% in Mbale city. Epidemiological information and laboratory test findings of the outbreaks in Kampala and Mbale cities during the study period are shown in Table 1. Four percent (15/318, 4%) of suspected cholera cases that were treated in Kampala city and eight percent (12/153) of the suspected cholera cases treated in Mbale city were non-residents who were on transit or had come for business and developed cholera. For these cases, no follow up was done to their places of origin or households. However, the respective districts of origin were alerted to take action. No household contacts listed and had received the cholera preventive package developed cholera.

**Table 1:** epidemiological description of the cholera outbreaks in Kampala and Mbale cities and laboratory test results of the stool samples, 2015-2016

Variable	Kampala city	Mbale city
**Epidemiological description**		
City population	1,561,696	507,588
Date outbreak started	29^th^ November 2015	6^th^ December 2015
Date of last confirmed cholera case	7^th^ January 2016	16^th^ January 2016
Total suspected cholera cases	318	153
Total suspected deaths	8	5
Total confirmed cases	58	41
Total confirmed cholera deaths	1	5
Case fatality rate (suspected cases)	2.5%	3.3%
Case fatality rate (confirmed)	1.7%	12.0%
Attack rate/1000 of the population (suspected cases)	0.20	0.30
Attack rate/1000 of the population (confirmed cases)	0.04	0.08
**Laboratory test results**		
No. of stool samples collected	282	97
No. of stool samples tested by culture	163	47
No. of positive samples for *V. cholerae* O1	58	41
No. of negative stool samples for *V. cholerae* O1	105	06
Percentage of *V. cholera* O1 positive stool samples	35%	87.2%

**Figure 2:**
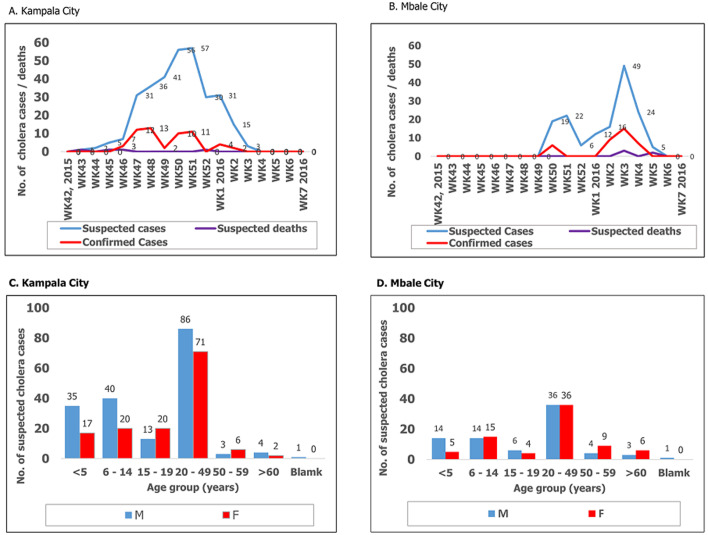
reported cholera cases and deaths and their age-sex distribution in Kampala and Mbale cities during the cholera outbreaks of 2015-2016: A) weekly reported cases and deaths in Kampala city; B) weekly reported cases and deaths in Mbale city; C) age-sex distribution in Kampala city; D) age-sex distribution in Mbale city

**Progression and age-sex distribution of the cholera outbreaks:** Kampala capital city was the first of the two cities to report cholera outbreak. The outbreak in Kampala city had three peaks while that in Mbale city had two peaks. The highest peak in Kampala city was during the 50^th^ week of 2015 and while the highest peak in Mbale city was in the 4^th^week of 2016. Kampala city reported the highest number of both suspected and confirmed cholera cases. There were no cholera cases reported among household contacts listed and given cholera preventive package. Laboratory test results were the basis of determining the end of the outbreaks. The outbreaks in both cities lasted for 6 weeks (41 days) and all ages and sexes were affected though not to the same extent. Reported cholera cases and deaths in the two cities and their age-sex distribution are shown in Figure 2. During these outbreaks statistically significant findings were noted only for the age group less than 14 years in Kampala city. The males were the most affected sex among the age groups less than 9 years and less than 14 years in Kampala City. The difference in cholera cases between the sexes in Kampala City were statistically significant, Chi-square statistic of 6.6894, p-value = 0.0097 at p < .05. There was no statistical difference between sexes in Mbale city for the age groups less 14 years; 78 males verses 75 females and the Chi-square statistic of 1.5132, p-value = 0.2187 (p values <0.05).

**Figure 3:**
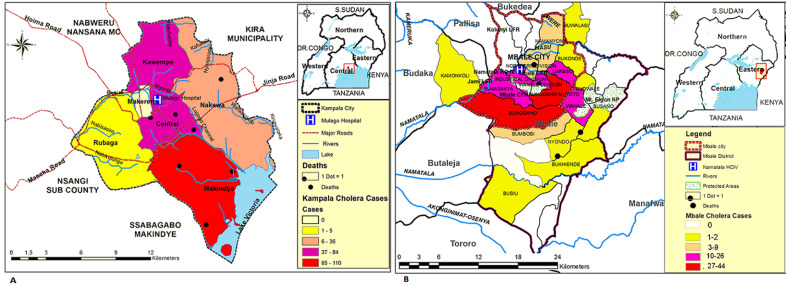
the map of the study area showing spatial distribution of cholera cases during cholera outbreaks in the two cities of Kampala and Mbale in 2015-2016; A) map of Kampala capital city showing distribution of cholera cases in the five municipalities; B) map of Mbale city showing distribution of cholera cases in the 13 sub divisions

**Spatial distribution of suspected cases in the two cities:** spatial distribution of cholera cases was non-uniform with majority of cases in the two administrative zones where the outbreaks started and shaded on the map as red and dark green coloured areas respectively as in Figure 3.

## Discussion

This study showed that by implementing cases and contacts centered strategy (3CS) as summarised in Figure 1 it is possible to rapidly control cholera outbreaks in cities with informal settlements, record fewer number of cholera cases and register no cholera cases amongst the households members of the cholera cases. Furthermore, by using this strategy, Kampala city reported 58 confirmed cholera cases among 318 cholera suspects and the outbreak lasted for 41 days. The numbers of cases reported in these outbreaks and the duration of the outbreaks were less than for cities in the sub-Saharan Africa region with similar settings (informal settlements). For instance higher numbers of cases were reported for cholera outbreaks in the following cities: Lusaka city, Zambia, where approximately 2,000 cases were reported [3]; Dar es Salaam, Republic of Tanzania, 3,371 cases with 36 deaths reported [26]; and Harare, Zimbabwe, 8,535 cases and 50 deaths were recorded [27]. Furthermore, the duration of some of the outbreaks in cities with informal settlement were much longer. Case in point are the cholera outbreaks in Kinshasa city, Democratic Republic of Congo where the outbreak lasted for almost two years [5]; Lusaka city, Zambia where the cholera outbreak was controlled after eight months (October 2017 to May 2018) [3]; Dar es Salaam, Tanzania, cholera outbreak lasted from 16^th^ August 2015-16^th^ January 2016 [26] and Harare, Zimbabwe, the outbreak lasted longer than 6 months in 2008 [4].

Most importantly, for all the household contacts that were listed and given cholera preventive package, none of them developed cholera or diarrhoea. Previously, a study in Bangladesh showed increased risk of cholera among household members of the cholera cases [28]. It should be noted that 3CS focussed on both cases and contacts at health facility and at household levels. The reasons for focusing on contacts at the household level were to prevent new infections among the contacts (household members) since they are important factor in cholera spread as previously documented [28]. Selective chemoprophylaxis which was a component of the package is safe use of the antibiotics by targeting persons with possible infection and having epidemiological linkage. There is another use of antibiotics known as mass chemoprophylaxis which has the potential to cause false confidence among the recipients and can easily lead to antibiotic resistance. During mass chemoprophylaxis, persons are given antibiotics irrespective of the epidemiological linkage to infections or risk/exposure. World Health Organization (WHO) does not recommend mass antibiotic chemoprophylaxis for cholera prevention [29]. Given that no new cholera cases were reported amongst the listed household contacts and outbreaks were controlled rapidly, we think that selective chemoprophylaxis which was one of the constituents of 3CS (Figure 1) could be employed in similar manner to oral cholera vaccines or other new initiatives to prevent cholera by complementing core cholera control interventions. However, further studies are needed to generate more specific information.

Surprisingly, though the study settings were urban areas where access to health care is expected to be high, community deaths resulting from persons who did not seek care were reported. We think that although the treatment is offered for free, some members in the community do not utilize the services provided. Therefore, the MOH will need to review the healthcare service delivery if Uganda is to meet the WHO roadmap for Global cholera elimination by 2030 [23]. In addition, some of the suspected cholera cases that were treated in both cities were non-residents that were on transit or on business trips. Henceforth, to prevent such scenario the local authorities of the two cities will need to strengthen food safety measures by targeting food handlers, vendors and eating places. The city authorities and the MOH will need to sensitize the communities through health education for disease prevention in two cities and in the immediate neighbourhoods.

**Strengths and weaknesses/limitations of the study:** the following were the strengths of the study: first, follow up of cholera cases to their homes ensured that cases were identified early and referred for appropriate treatments which is important in reducing the case fatality rate and limiting infection spread. Second, selective chemoprophylaxis by health workers resulted in monitored use of antibiotics which is important to avoid antimicrobial resistance. Third, the health workers got clear understanding of the risk factors responsible for the outbreaks by reaching the homes of the suspects and those in their neighbourhoods. Forth, there were community deaths that were identified and the households were given the preventive packages. This had the potential of preventing new cholera infections and disease spread resulting from the handling of the dead bodies [30]. Fifth, many persons with diarrheal which is a major cause of morbidity and mortality in the informal settings [31] were identified and measures for diarrhea prevention instituted. Finally, the strengths of this study were also noticed by the technical leadership of the MOH which consequently cleared the revision of the old national cholera guidelines to include the new findings. The revised Uganda national cholera prevention and control guidelines document is available and accessible [32].

**Weaknesses/limitations of this study:** first, though, this study demonstrated rapid outbreak control and less number of reported cholera cases as a result of implementation of 3CS than those of other cities in sub-Saharan Africa with informal settlements [3,33], this was not the most appropriate method. Therefore, further studies such as the case-control studies which show cause-effect relationships are needed. Second, we could not determine why more males than females for the ages less than 14 years were affected in Kampala city than the female counterparts. Further studies are also recommended to provide more information on why more males in the age groups less than 14 years were the most affected sex group in Kampala city. Third, the attack rate for Mbale city could be less than actual since the population used to compute this value included sub counties in Mbale district (outside the Mbale city) that were also reporting cholera cases. However, since cholera cases from these sub counties were also included under Mbale city we think that the effect of this was minimal and did not significantly affect the final attack rate value.

## Conclusion

This study showed that by implementing 3CS, it was possible to rapidly control the cholera outbreaks in cities, identify community cases and deaths and register a smaller number of cholera cases in the two cities (Kampala and Mbale) that had informal settlements. There were also no cholera cases reported amongst persons (household contacts) listed and given cholera preventive package. Selective chemoprophylaxis could complement core cholera control interventions in similar manner to oral cholera vaccines or other new initiatives. These findings could be put to use to control cholera outbreaks in cities with similar settings (informal settlements), however, further studies such as case control studies are needed to guide such rollout.

### What is known about this topic

- Cholera is an intestinal disease that is preventable and treatable. However, it is a major cause of morbidity and mortality in many countries in sub-Saharan Africa;
- Cholera occurs when a person consumes food or drinks contaminated with V. choleraeorganisms and cases tend to occur in household contacts of the cholera cases;
- Treatment of the cholera cases using appropriate antibiotics kills the V. choleraepresent in the intestines or stools of the cases.

### What this study adds

- In order to rapidly control cholera outbreaks in cities with informal settlements a comprehensive approach that targets both cholera cases and their household members (contacts) such as 3CS is required;
- Selective antibiotic chemoprophylaxis, a component of 3CS could be employed in similar manner to oral cholera vaccines to prevent cholera by complementing core cholera control interventions;
- Even in urban areas (cities) with no limiting factor of physical access to health facilities and with free treatment offered by the government, community deaths from cholera can still occur in significant numbers.

## References

[1] Deen J, Mengel MA, Clemens JD (2020). Epidemiology of cholera. Vaccine.

[2] WHO (2018). Cholera-Zimbabwe: disease outbreak news, 20 September 2018 - Zimbabwe. ReliefWeb.

[3] Sinyange N, Brunkard JM, Kapata N, Mazaba ML, Musonda KG, Hamoonga R (2018). Cholera Epidemic-Lusaka, Zambia, October 2017-May 2018. MMWR Morb Mortal Wkly Rep.

[4] Chambers K (2009). Zimbabwe´s battle against cholera. Lancet.

[5] Bompangue D, Moore S, Taty N, Impouma B, Sudre B, Manda R (2020). Description of the targeted water supply and hygiene response strategy implemented during the cholera outbreak of 2017-2018 in Kinshasa, DRC. BMC Infect Dis.

[6] Bwire G, Ali M, Sack DA, Nakinsige A, Naigaga M, Debes AK, Ivers LC (2017). Identifying cholera “hotspots” in Uganda: an analysis of cholera surveillance data from 2011 to 201. PLoS Negl Trop Dis.

[7] Mbonye A, Wamala J, Kaboyo W, Tugumizemo V, Aceng J, Makumbi I (2012). Repeated outbreaks of viral hemorrhagic fevers in Uganda. Afr Health Sci.

[8] Bwire G, Malimbo M, Maskery B, Kim YE, Mogasale V, Levin A, Ryan ET (2013). The Burden of Cholera in Uganda. PLoS Negl Trop Dis.

[9] Kwesiga B, Pande G, Ario AR, Tumwesigye NM, Matovu JKB, Zhu BP (2017). A prolonged, community-wide cholera outbreak associated with drinking water contaminated by sewage in Kasese District, western Uganda. BMC Public Health.

[10] Sauvageot D, Njanpop-Lafourcade B-MM, Akilimali L, Anne JCC, Bidjada P, Bompangue D (2016). Cholera incidence and mortality in sub-Saharan African sites during multi-country surveillance. PLoS Negl Trop Dis.

[11] Bwire G, Munier A, Ouedraogo I, Heyerdahl L, Komakech H, Kagirita A, Ryan ET (2017). Epidemiology of cholera outbreaks and socio-economic characteristics of the communities in the fishing villages of Uganda: 2011-2015. PLoS Negl Trop Dis.

[12] Bwire G, Malimbo M, Makumbi I, Kagirita A, Wamala JF, Kalyebi P (2013). Cholera surveillance in Uganda: an analysis of notifications for the years 2007-2011. J Infect Dis.

[13] Oguttu DW, Okullo A, Bwire G, Nsubuga P, Ario AR (2017). Cholera outbreak caused by drinking lake water contaminated with human faeces in Kaiso Village, Hoima District, Western Uganda, October 2015. Infect Dis Poverty.

[14] Bwire G, Sack DA, Kagirita A, Obala T, Debes AK, Ram M (2020). The quality of drinking and domestic water from the surface water sources (lakes, rivers, irrigation canals and ponds) and springs in cholera prone communities of Uganda: an analysis of vital physicochemical parameters. BMC Public Health.

[15] Pande G, Kwesiga B, Bwire G, Kalyebi P, Riolexus A, Matovu JKB (2018). Cholera outbreak caused by drinking contaminated water from a lakeshore water-collection site, Kasese District, south-western Uganda, June-July 20 PLoS One.

[16] Okello PE, Bulage L, Riolexus AA, Kadobera D, Kwesiga B, Kajumbula H (2019). A cholera outbreak caused by drinking contaminated river water, Bulambuli District, Eastern Uganda, March 20. BMC Infect Dis.

[17] Ministry of Health Uganda (2007). Prevention and control of cholera, operational guidelines for the district health workers and planners.

[18] George CM, Hasan K, Monira S, Rahman Z, Saif-Ur-Rahman KM, Rashid M, Ryan ET (2018). A prospective cohort study comparing household contact and water Vibrio cholerae isolates in households of cholera patients in rural Bangladesh. PLoS Negl Trop Dis.

[19] Legros D, McCormick M, Mugero C, Skinnider M, Bek´obita DD, Okware SI (2000). Epidemiology of cholera outbreak in Kampala, Uganda. East Afr Med J.

[20] Alajo SO, Nakavuma J, Erume J (2006). Cholera in endemic districts in Uganda during El Niño rains: 2002-2003. Afr Health Sci.

[21] MacNeil A, Shoemaker T, Balinandi S, Campbell S, Wamala JF, McMullan LK (2012). Reemerging Sudan Ebola virus disease in Uganda, 2011. Emerg Infect Dis.

[22] Goeijenbier M, van Kampen JJA, Reusken CBEM, Koopmans MPG, van Gorp ECM (2014). Ebola virus disease: a review on epidemiology, symptoms, treatment and pathogenesis. Neth J Med.

[23] World Health Organization (2017). Ending cholera: aglobal roadmap to 2030. Glob Task Force Cholera Control.

[24] Uganda Bureau of Statistics (2006). Compendium of Statistical Concepts and defitions used in the Uganda Statistical System and Services.

[25] Ministry of Health Knowledge Management Portal. Village Health Team Strategy and Operational Guidelines.

[26] McCrickard LS, Massay AE, Narra R, Mghamba J, Mohamed AA, Kishimba RS (2017). Cholera mortality during urban epidemic, Dar es Salaam, Tanzania, August 16, 2015-January 16, 2016. Emerg Infect Dis.

[27] (2018). World Health Organization Cholera - Zimbabwe.

[28] Weil AA, Khan AI, Chowdhury F, Larocque RC, Faruque ASG, Ryan ET (2009). Clinical outcomes in household contacts of patients with cholera in Bangladesh. Clin Infect Dis.

[29] World Health Organization (2018). Technical note use of antibiotics for the treatment and control of cholera May 2018 indications for antibiotic use.

[30] Sack RB, Siddique AK (1998). Corpses and the spread of cholera. Lancet.

[31] Patel RB, Stoklosa H, Shitole S, Shitole T, Sawant K, Nanarkar M (2013). The high cost of diarrhoeal illness for urban slum households-a cost-recovery approach: a cohort study. BMJ Open.

[32] Ministry of Health, Uganda (2017). Prevention and control of cholera: operational guidelines for the national and district health workers & planners control of diarrhoeal diseases (CDD) Section Community Health Department, Ministry of Health.

[33] Mason PR (2009). Zimbabwe experiences the worst epidemic of cholera in Africa. J Infect Dev Ctries.

